# What do beginning students, in a rurally focused medical course, think about rural practice?

**DOI:** 10.1186/s12909-016-0829-4

**Published:** 2016-12-07

**Authors:** Louise Young, Daniel B. Lindsay, Robin A Ray

**Affiliations:** 1 College of Medicine and Dentistry James Cook University, Townsville, 4811 Australia; 2 Department of Public Health and Tropical Medicine, College of Public Health, Medical and Veterinary Sciences James Cook University, Townsville, 4811 Australia

**Keywords:** Selection, Medical student perceptions, Urban-rural origin students, Rural recruitment, Rural doctor, Rural life

## Abstract

**Background:**

Medical schools may select students for their attitudes towards rural medical practice, yet the rural–urban disparity in availability of medical practitioners and services has not diminished in recent times despite government initiatives and increasing numbers being trained for a career in medicine. One medical school, with a focus on rural and remote medicine, aims to select students with positive perceptions for rural medical practice. A research project collected data on the perceptions of these medical students in the first week of their medical studies.

**Methods:**

Students completed a low stakes essay on the life and work of a rural doctor. Initially, this formed part of a literacy assessment to determine any students requiring remediation. All students were asked if they would consent to their essay being reviewed for a research project.

Data was obtained from those students who consented and handed their essays in for review. The 103 student essays underwent thematic analysis and sentences were coded into three main themes of rural lifestyle, doctor role and rural practice. Second level themes were further elicited and results were quantified according to whether they were positive or negative. Positive themes included rural lifestyle, doctor role, views of doctor, impact on community, broader work and skills knowledge, and better relationships with community and patients. Negative themes included doctor’s health, pressure on doctor, family problems, greater workload, privacy and confidentiality issues, cultural issues, isolation, limited resources and financial impacts. Quantitisation of this data was used to transform essay sentences into a numerical form which allowed statistical analysis and comparison of perceptions using Z tests.

**Results:**

No significant differences on the number of positive and negative responses for rural lifestyle and rural practice were found. The rural doctor role had a significantly more positive than negative views. Significant differences were found for positive views of the rural doctor role and negative views of rural practice. Participants from a capital city background reported a significantly higher percentage of responses related to negative views of rural practice than their regional and rural counterparts. Students from capital city areas had significantly more negative views about the rural doctor role, especially related to workload, limited resources and isolation than students from rural and regional areas.

**Conclusion:**

Students entering medical school already have both positive and negative views about the life and work of a rural doctor. Those students from capital city areas have significantly more negative views despite being selected to enter a medical course with a rural focus based on their expressed rural perceptions. Further work is required to refine selection criteria and the year level experiences and learning opportunities which may positively influence student perceptions about rural medical practice to overcome early negative perceptions at the beginning of medical school.

## Background

Despite the high ratio of medical doctors per head of population in Australia (201.9 GPs and 117.9 specialists per 100000 persons) [1], 34% of Australians residing outside major urbanised areas [2] have access to less than half the number of medical practitioners when compared with people living in metropolitan areas [3, 4]. A similar maldistribution is evident in the United States of America where 11% of the nation’s doctors work rurally to serve 20% of the population [5]. Large areas of both countries are medically underserved, identifying a need for medical students to be trained specifically to work as doctors in rural and remote locations [6].

Medical schools in Australia are now training double the number of students compared with ten years ago [7]. These have included the compulsory 25% of places in each medical school for bonded medical places which require students to undertake the course equivalent time as return of service in underserved areas [8]. Yet, the initiatives of increased medical schools and rural bonded students have not closed the rural to urban medical services gap.

The rural/urban medical practitioner maldistribution was a key driver for the establishment of a regional medical program in 2000 which aims to prepare graduates who can work as doctors in rural and remote locations and includes a minimum of 20 weeks rural and remote medicine [9]. Student selection includes a written application and interview to identify personal characteristics and attitudes consistent with rural practice. The course overview also informs applicants that throughout the course they will experience a minimum of 20 weeks of rural and remote medicine.

This project is phase one of a longitudinal research project investigating whether a medical program with a rural, underserved focus has an impact on student perceptions and career intentions for rural practice over time. Few studies have investigated the stability of longitudinal personal perceptions related to working in a health profession. Given the competitive nature of selection for medical programs, are intentions stated in the application process always genuine? To assess rural perceptions and interest in rural medicine among beginning medical students, a low stakes investigation was required.

## Methods

The aims of this study were to:

- □ Identify themes related to medical student perceptions about the life and work of a rural doctor.
- □ Compare and contrast themes in medical student essays about the life and work of a rural doctor.

### Recruitment and data collection

In 2012 and 2013 all medical students entering the first year of a six year, undergraduate medical program at James Cook University were required to complete an academic essay with a defined topic of the life and work of a rural medical doctor, as part of a routine literacy assessment. On completion of the task, students were invited to submit their essays as the primary data for analysis, as part of this research project.

It was believed that by asking students to write about the life and work of a rural doctor, after they had a place in a medical program, would avoid interview response bias often found in the selection process [10]. These students were already students in a medicine program and as the essay was for literacy assessment purposes and not used for summative grading, it was considered a low stakes assessment. As it was a low stakes assessment it was felt students would be less inhibited and would feel free to write anything about the set topic. Additionally, as personal statements have been used to assess attributes and attitudes in previous studies, assessment of these writing pieces would reveal student attitudes and opinions concerning rural medical practice that were not expressed during selection [11, 12].

### Data analysis

Data collected in week one of the first year of the Bachelor of Medicine Bachelor of Surgery course and submitted by consenting students underwent a three level qualitative analysis [13]. Confirmability of the analysis was enhanced through shared coding sessions and theme generation among researchers, with consensus used to resolve any discrepancies. Full details of this template analysis and the themes generated have been reported in an earlier paper [14].

A quantizing approach to qualitative data was used to further analyse the content of the medical student themes elicited from the essays [15]. Quantitising refers to numerical translation, transformation or conversion of qualitative data and is a process for making judgements about similarities and differences. By converting qualitative data to numerical data we are able to interact with and analyse the data in different ways.

Quantitizing was used to transform essay sentences into a numerical form which allowed for further comparison and analysis [15]. Student sentences within each theme and sub-theme were classified by two researchers for reliability. Sentences representing either positive or negative perceptions of rural life and rural medical practice and totals were quantified as percentages to provide an indication of their relative importance and occurrence. These positive and negative perceptions of rural practice were then analysed according to whether respondents were from an urban or rural background. As there were different numbers of positive and negative responses, z-tests for two population proportions examined significant differences in numbers of positive and negative responses for each major theme. The z-test for two population proportions was performed because the samples being compared where disproportionate, which needed to be taken into account when considering the differences between groups.

To compare individual perceptions about rural practice within the cohort, basic demographic data was collected from the student database and this information was used as variables in the quantitative analysis. Background was determined by home postcode with rural background being defined as having completed five or more years of education in a rural location as classified by the Australian Standard Geographical Classification – Remoteness Areas (ASGC-RA) system [16]. Classifications are based on population size and the distance of a location from the nearest urban centre. ASCG-RA 1 equates to major cities, while ASGC-RA 2-5 covers inner/outer regional, remote and very remote Australia. As nearly 70% of the Australian population lives in ASGC-RA 1, it was decided to have major cities as one category and other ASGC-RA categories combined as the other category.

### Participants

A total of 103 students contributed their essays with those from 12 international students being excluded from the rural-urban analysis. This left 91 (46 male and 45 female) students. Twenty-four were urban background (ASGC-RA = 1), while 67 were regional or rural background (ASGC-RA = 2+). The urban/rural context of the international students (n = 12) background was unknown and therefore their 81 responses were excluded from the rural/urban analyses. Demographic information was not obtained for six participants and their responses were not included in the analyses for demographic information.

Ethics approval for this project was obtained from James Cook University Human Research Ethics Committee, (H6096). Consent was presumed when students voluntarily submitted their writing exercise through a secure assignment box set up purposely for this project.

## Results

### Major themes

The 103 respondents provided 629 statements relating to perceptions about rural medical practice. A Chi-square analysis of total participant responses found no significant difference in number of positive (*n* =330, 52.5%) or negative (*n* = 299, 47.5%) responses given by participants (*χ* ^2^ = 1.53, *p* < .05). This suggests that perceptions surrounding rural practice are relatively mixed in first year medical students. Themes and sub-theme percentages and representative statements from students are shown in Table 1.

**Table 1:** Major themes and sub-themes and representative statements

Major themes	*N* (%) of responses	Sub-themes	*N* (%) of responses	Representative statements
Rural lifestyle: positive statements	15 (2.4%)		15 (2.4%)	*“…more relaxed country atmosphere allows many doctors to greatly enjoy their down time.”*
Rural lifestyle: negative statements	25 (4%)		25 (4%)	*“Rural doctors coming from larger cities will have to sacrifice the lifestyle they are used too, including technological advantages and reliance on co-workers and partners.”*
Doctor role: positive statements	78 (12.4%)			
		Positive impact on the community	22 (28.2%)	*“Doctors have a chance to make a remarkable impact on a community and drastically improve it.”*
		Educator and communicator	15 (19.2%)	*“The rural doctor must act as a form of communication, an advocate, educating these remote communities about good health and techniques needed to be done in order to be healthy and to minimize chances of illness.”*
		Positive views of rural doctor	41 (52.6%)	*“Rural doctors are not just another member of their community, they are the cornerstone.”*
Doctor role: negative statements	56 (8.9%)			
		Doctor’s health	20 (35.7%)	*“Long hours, a variety of abnormal cases and needs of particular communities can have a detrimental toll on rural doctor’s health.”*
		Pressure on doctor	20 (35.7%)	*“In some towns there may only be one doctor and this may place pressure on the doctor…”*
		Family problems	16 (28.6%)	*“This can make it hard to separate work and life for rural doctors and may cause strain on family life.”*
Rural practice: positive	206 (32.7%)			
		Broader work/skills/knowledge	95 (46.1%)	*“…practitioners most likely will come across various symptoms and illness that are less frequently seen in more modernized cities, perhaps due to the alternative environment and culture rural areas offer.”*
		Better relationships with patients/community/co-workers	111 (53.9%)	*“…opportunity to see patients from the beginning to the end of their treatments, forming bonds with the community members and gaining respect and satisfaction.”*
Rural practice: negative	249 (39.6%)			
		Greater workload	50 (20.1%)	*“…work load for rural doctors is greater, as not only do they have patients to visit but their work does not end once they leave the hospital.”*
		Privacy/confidentiality issues	39 (15.7%)	*“…patients will know where the doctor lives and even visit the doctor’s house if they have a medical problem.”*
		Cultural issues	18 (7.2%)	*“The cultural differences can create difficulties in the doctor’s ability to treat a given patient.”*
		Isolation	64 (25.7%)	*“One of the biggest challenges a rural doctor faces is often the extreme isolation or remoteness of the community they are in…”*
		Limited resources	77 (30.9%)	*“A rural doctor will have limited access to health resources when compared to their urban counterparts.”*
		Financial	1 (0.4%)	*“…rural doctors may have less opportunity to make larger salaries.”*

Table 1 also presents responses and percentages for positive and negative responses. No significant differences between positive and negative responses for rural lifestyle and views of rural practice were found (*p* > .05). There was a significant difference between the number of positive and negative views of the rural doctor role (*z* = 2.79, *p* < .01) with students reporting more positive views.

### Rural background

A summary of ASGC-RA1 and ASGC-RA2+ origin responses (*n* = 541) is shown in Table 2. As ASGC-RA 1 (*n* = 150) and ASGC-RA 2+ (*n* = 391) provided different numbers of total responses, a z-test for two population proportions examined any significant differences in responses based on rural background.

**Table 2:** Responses for each major theme based on student ASGC-RA background

		ASGC-RA1^a^		ASGC-RA2-5^b^	
		Number of responses	% of total responses	Number of responses	% of total responses
Rural Lifestyle	Positive	6	4	18	4.6
	Negative	4	2.7	10	2.6
Rural Doctor Role	Positive	10	6.7	57	14.6
	Negative	17	11.3	35	9
Rural Practice	Positive	42	28	135	34.5
	Negative	71	47.3	136	34.7
Total Responses		150	100	391	100

^a^Australian Standard Geographical Classification – Remoteness Area 1 = major cities

^b^Australian Standard Geographical Classification – Remoteness Areas 2–5 = inner/outer regional, remote and very remote Australia

Significant differences were found in number of responses for positive views of the rural doctor role (*z* = -2.51, *p* < .05) and negative views of rural practice (*z* = 2.69, *p* < .01). Participants from an urban background reported a significantly higher percentage of responses expressing negative views about rural practice and a significantly lower percentage of responses related to positive views of the rural doctor, than their regional and rural counterparts. No other significant differences were found (all *p* > .05).

The results suggest that, even in entry level medical students, there are some differences in perceptions related to rural lifestyle, rural doctor role and rural practice which are influenced by whether students have been raised in urban ASGC-RA 1 areas or regional/rural ASGC-RA 2+ areas. Of interest is whether these perceptions are able to be changed over time following exposure to medical training comprising repeated, rural clinical training experiences.

## Discussion

Despite being enrolled in a rurally focussed medical course, beginning students varied widely in their perceptions of rural medical practice. Location, organisation, selection methods and curriculum have been shown to influence choice of specialty and location of practice for medical students [14, 17, 18]. Our study concurs with location of origin being an important factor as students from regional and rural backgrounds in this cohort were more positive and realistic in their descriptions of rural practice [14]. Yet, those students with largely negative perceptions remain. Selection processes may have a role to play in answering this finding by employing methods which will identify those students who are ‘faking good’ during the selection process [17].

Based on the mission of our medical school our selection criteria aims to identify applicants with an interest in rural medicine and it is expected that applicants with these attributes gain entry to JCU. However, studies have shown that the high stakes nature of medical selection may induce some applicants to present misleading information during their selection interview [17]. Thus, some students entering a course focused on rural and remote medicine may not have an interest in rural practice, but be highly motivated by their need to study medicine regardless of the focus of the medical course.

### Implications for medical education

The results show that the perceptions of beginning medical students about the work of a rural doctor are relatively mixed which may differ from their stated opinions during medical selection processes. However, even among entry level medical students, perceptions of rural lifestyle, rural doctor role and rural practice are influenced by whether students have urban ASGC-RA 1 or regional/rural ASGC-RA 2+ background. Our results show that within this cohort of students those with an urban upbringing generated more negative views about rural practice than students with a rural or regional upbringing. Of interest is if and when these perceptions are changed following immersion in a rural focused medical curriculum and repeated, rural clinical training experience.

Curriculum and rurally based clinical opportunities have been found to positively influence perceptions of rural practice [19, 20]. Current JCU graduate outcomes indicate rural experiences have a positive impact on both intent and reality of practising in rural locations. At graduation, 88% of JCU graduates intend to practise outside capital cities compared with 31% of graduates from other medical schools, 67% undertook internship outside a metropolitan centre and 47% in outer regional centres compared with 17% and 5% respectively of graduates from other Australian medical schools [21]. Other graduate outcomes indicate that early career rural medical practice is enhanced by education strategies including repeated rural clinical placements across several years of the MBBS program, rural internship opportunities during medical school, supportive rural general practice experiences and selecting applicants with rural backgrounds [18, 21, 22]. However, over time the selection of more rural origin medical students has not changed overall student perceptions about rural practice [18, 19, 23, 24].

Outcomes from this study indicate urban students show less rural interest which reinforces that medical schools need to develop rural training experiences to foster greater acceptance of rural practice as a potential life and work location. Based on the number of negative views raised by participants in this sample, attention needs to be directed at overcoming the perception of isolation and greater workload for rural doctors. Particular focus may need to be directed to those with an urban background who may require specific rural knowledge and experiences which are already common knowledge for rural origin students.

Early mixed perceptions about the life and work of a rural doctor may be ameliorated by repeated, positive and longitudinal rural clinical placements. Previous negative perceptions can be transformed positively, especially for those students originating from ASGC-RA 1 [22, 25]. It is expected that many rural origin students will return to regional/rural areas to practise, however the conversion of ASGC-RA 1 students into medical graduates, who choose to work in regional and rural Australia, is a net gain for the provision of practising clinicians to these underserved populations and is helping to address medical workforce maldistribution^6^. This study has identified urban students begin with negative perceptions about rural practice, yet something changes this perception as they progress through medical school.

### Limitations

Results may have been influenced by a number of limitations. As students knew their paper was not for summative assessment they may have not been honest in their writing. However, a study by Fischer et al found no difference in themes addressed or levels of reflection when students completed work online in a blog, with less formality required, when compared with a traditional essay [26]. As this was a low stakes assessment, students may have actually been more honest and forthright in their description and analysis of the life and work of a rural doctor. Students also volunteered to hand in their essay for inclusion as data in this project. It may be that only those particularly engaged students volunteered to hand in their essays for analysis. However, as essays from two consecutive cohorts were analysed, we are confident that the results reflect accurate rural perceptions for these students.

Future investigations following students longitudinally to ascertain changes in perception and determine critical educational experiences and rural placements that impact on student perceptions, is warranted. It would also be interesting to ask final year medical students to write the same essay after nearly six years of a rural orientated curriculum to determine the stability of student perceptions across time and rural clinical experiences.

The advantage of this work is that baseline information has been collected using a low stakes assessment, and while statements made by these first year medical students may differ from what was stated in their selection application, the nature of this low stakes essay is more likely to have elicited honest perceptions. The timing and context of this study has elicited accurate perceptions and avoided desirability bias that occurs in high stakes settings such as medical selection interviews.

## Conclusion

Despite a selection process that favours individuals with a positive rural outlook, our findings have shown that first year medical students’ perceptions about the life and work of a rural doctor are relatively mixed, with those from an urban background being more negative than those from regional or rural backgrounds. Findings from this project suggest the pressure for a place in medicine is so strong, that during selection processes, some applicants may present positive rural perceptions irrespective of their true perceptions. A low stakes essay on a topic about rural life and rural medical practice, which was thematically analysed, is evidence this has occurred for many medical students. This was especially evident for students from capital city origins. Further work is required to refine selection criteria and the year level experiences and learning opportunities which may positively influence student perceptions about rural medical practice to overcome early negative perceptions at the beginning of medical school.
